# Draft genome sequencing of a *Staphylococcus aureus* clinical isolate from an inflamed wound

**DOI:** 10.1128/mra.00100-26

**Published:** 2026-05-18

**Authors:** Ibrahim A. Ahmed, Zaid A. Thabit, Arif A. Ghayyib, Yaseen I. Mamoori, May R. Jaafer, Nisreen S. Hasan

**Affiliations:** 1 Applied Biology Department, College of Biotechnology, Al-Nahrain University 54645, Baghdad, Iraq; 2 Biotechnology Research Center, Al-Nahrain University 54645, Baghdad, Iraq; 3 Faculty of Medical Sciences, Jabir Ibn Hayyan University for Medical and Pharmaceutical Sciences, Najaf, Iraq; 4 Department of Molecular and Medical Biotechnology, College of Biotechnology, Al-Nahrain University 54645, Baghdad, Iraq; 5 Department of Forensic Biology, Higher Institute of Forensic Sciences, Al-Nahrain University 54645, Baghdad, Iraq; 6 Department of Anesthesia Techniques, College of Health and Medical Techniques, Al-Esraa University 554706, Baghdad, Iraq; University of Pittsburgh School of Medicine, Pittsburgh, Pennsylvania, USA

**Keywords:** *Staphylococcus aureus*, Al-Kadhimiya Hospital, whole genomic sequencing, ST1

## Abstract

In this study, we isolated *Staphylococcus aureus* (SA8-IRQ) from a patient suffering from an inflamed wound at Al-Kadhimiya Hospital in Baghdad. The whole genome of the bacteria was sequenced and analyzed. It is 2.8 Mb in size, has 32.7% GC content, and contains several virulence and antimicrobial genes.

## ANNOUNCEMENT

*Staphylococcus aureus* is a gram-positive, round-shaped, opportunistic pathogenic bacterium. It is found on the human and animal bodies as part of the normal flora, but under specific conditions, it can cause various diseases (1, 2). *Staphylococcus aureus* is among the most prevalent bacterial species, known for its high potential to develop antibiotic resistance, including methicillin-resistant *S. aureus* (MRSA) (3).

Just a few years after penicillin was first introduced in clinical use in hospitals, *S. aureus* acquired resistance to the antibiotic, making it the first bacterium to show antibiotic resistance (4). The development of antibiotic resistance poses a serious threat to human health and represents a significant global challenge (5). Several factors increase the pathogenicity of *S. aureus*, which produces numerous virulence factors, including immunomodulators, adhesins, and toxins (6–8).

The bacterial isolate was obtained from a human patient with an inflamed wound and was cultivated on mannitol salt agar and incubated at 37°C for 24 h. Then, a single colony was picked and incubated in brain heart infusion (BHI) broth. The isolate was identified by the VITEK 2 system and by molecular detection using PCR targeting the *S. aureus* thermonuclease gene (nuc) and sequencing (9). Then, the *S. aureus* isolate was stored in 25% glycerol at −20°C.

The bacterial isolate was cultivated in BHI broth at 37°C with shaking at 150 rpm under aerobic conditions overnight. Then, genomic DNA was extracted using the Wizard Genomic DNA Purification Kit (Promega, USA), and DNA concentration and purity were tested using the Nanodrop 2000 UV-Vis Spectrophotometer (Thermo Fisher Scientific, USA). DNA integrity was assessed by agarose gel electrophoresis, and the DNA was then sent to Apical Scientific (Selangor, Malaysia) for sequencing.

The sequence libraries (~500 bp) were generated using the VAHTS Universal Plus DNA Library Prep Kit for Illumina (No. ND801-C5, Vazyme Biotech Co., Ltd., China) and sequenced on the Illumina NovaSeq X Plus platform (Illumina, USA), yielding 2 × 150 bp paired-end reads. A total of 13,990,018 reads (6,995,009 reads per end) were generated. The data quality was evaluated by FastQC (v0.12.1) (10) and fastp (v0.24.0) (11). Filtered reads were assembled using Unicycler (v0.5.1) (12), which internally employs SPAdes. Quast (v5.3.0) (13) and CheckM2 (v1.1.0) (14) were used to assess genome assembly quality. MLST (v2.22.0) (15) was used for sequence typing of the isolate.

The *S. aureus* SA8-IRQ genome size was 2,809,488 bp, and the GC content was 32.7%. The genome assembly consisted of 17 contigs, with an N50 of 516,658 bp and an L50 of three contigs. The genome was functionally annotated using NCBI Prokaryotic Genome Annotation Pipeline (v6.10) (16), which predicted 2,670 coding sequences, 56 tRNAs, 4 rRNAs, and 4 ncRNAs (Table 1).

**TABLE 1:** Genome features of *Staphylococcus aureus* SA8-IRQ

Feature	Value
Genome size (bp)	2,809,488
No. of contigs	17
GC content (%)	32.7
N50 (bp)	516,658
L50 (no. of contigs)	3
Fold coverage (×)	721.70
Genes (total)	2,805
Coding sequences (total)	2,741
Gene (coding)	2,670
Genes (RNA)	64
rRNAs	4
tRNAs	56
ncRNAs	4
Completeness (%)	100
Sequence typing	ST1
Resistance genes	mecA (methicillin), blaZ, blaI, blaR1 (beta-lactam), tet(K), tet(38) (tetracycline), erm(C) (macrolide/lincosamide/streptogramin), ant (6)-Ia (aminoglycoside), sat4 (streptothricin), aph(3′)-IIIa (aminoglycoside/kanamycin), mepA (multidrug efflux), lmrS (multidrug MFS transporter), cadD (cadmium resistance), pbp4 (cephalosporin resistance)
Virulence factors	Gamma-hemolysins (hlgA/B/C), leukocidins (lukD/E), intercellular adhesin biosynthesis/export protein (IcaC), zinc metalloproteinase aureolysin, serine proteases SplA and SplB, staphylokinase, delta-hemolysin, complement inhibitor SCIN-A, staphylococcal enterotoxin (seh)

**Fig 1:**
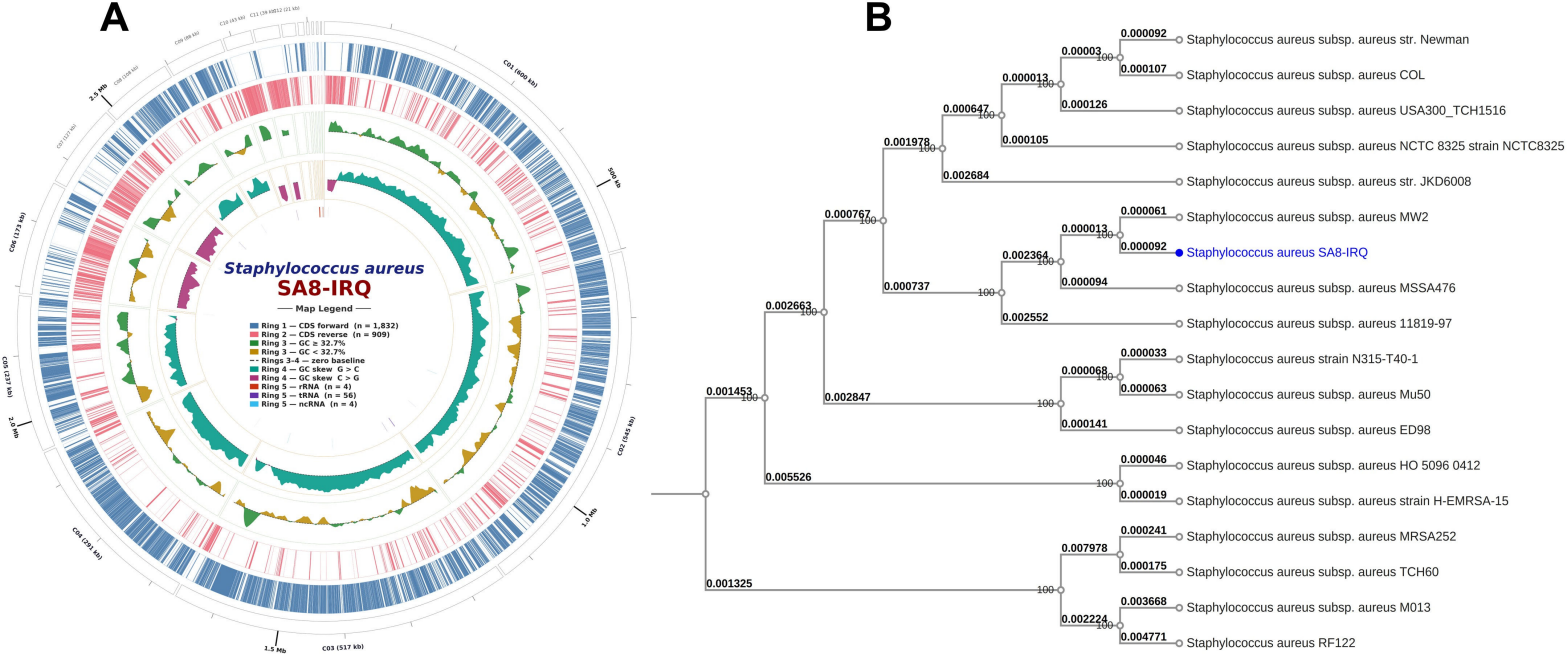
Circular genome map and phylogenetic analysis of *Staphylococcus aureus* SA8-IRQ. (**A**) Circular genome map of the draft genome (17 contigs; 2,809,488 bp). The map was generated using pyCirclize v1.10.1 (17). (**B**) The phylogenetic tree was generated using the Codon Tree method within the BV-BRC platform based on the alignment of 1,000 single-copy core genes using the RAxML algorithm (18). Branch lengths represent the number of substitutions per site (genetic distance), and node labels indicate bootstrap support values (100 replicates). SA8-IRQ (blue) clustered with strains MW2 and MSSA476, consistent with sequence type ST1.

A circular genome map (Fig. 1A) was generated using pyCirclize v1.10.1 (17). Phylogenetic relationships were inferred using the Codon Tree method within the BV-BRC platform, employing the RAxML algorithm (18) with 100 bootstrap replicates. Seventeen *S. aureus* reference genomes were included as comparators, representing major clinically and epidemiologically relevant lineages of both MRSA and methicillin-susceptible *S. aureus* strains with publicly available genome sequences in NCBI (Fig. 1B). Antimicrobial resistance genes and virulence factors were identified (Table 1) using AMRFinderPlus (v3.12.8) (19).

## Data Availability

The draft genome sequence of *Staphylococcus aureus* strain SA8-IRQ has been deposited in NCBI GenBank under accession number JBTLFM000000000.1 (BioProject: PRJNA1394457, BioSample: PRJNA1394457, BioSample: SAMN54320989, Assembly: GCF_054461255.1). The raw paired-end Illumina reads are available in the Sequence Read Archive under accession number SRX31621181.
